# Association between diuretic use and the risk of vertebral fracture after stroke: a population-based retrospective cohort study

**DOI:** 10.1186/s12891-019-2471-x

**Published:** 2019-03-04

**Authors:** Shu-Man Lin, Shih-Hsien Yang, Chih-Yung Wang, Huei-Kai Huang

**Affiliations:** 1 Department of Physical Medicine and Rehabilitation, Buddhist Tzu Chi General Hospital, No. 707, Sec. 3, Chung Yang Rd., Hualien, 97002 Taiwan; 2 School of Medicine, Tzu Chi University, No.701, Sec. 3, Chung Yang Rd., Hualien, 97002 Taiwan; 3 Department of Family Medicine, Buddhist Tzu Chi General Hospital, No. 707, Sec. 3, Chung Yang Rd., Hualien, 97002 Taiwan

**Keywords:** Stroke, Vertebral fracture, Diuretics, Thiazides, Loop diuretics, Cohort study

## Abstract

**Background:**

Stroke is a major risk factor for osteoporosis and fractures. No study has evaluated the association between diuretic use and risk of vertebral fracture in stroke patients, although a considerable proportion of stroke patients are prescribed diuretics for hypertension. Our study aimed to investigate whether treatment with thiazides or loop diuretics affects the risk of vertebral fracture after stroke.

**Methods:**

A population-based propensity score-matched retrospective cohort study was conducted using the Taiwan National Health Insurance Research Database. Patients with a new diagnosis of stroke between 2000 and 2011 were included. After propensity score matching, 9468 patients were included in the analysis of the effect of thiazides, of who 4734 received thiazides within 2 years after stroke. To analyze the loop diuretic effect, 4728 patients were included, of who 2364 received loop diuretics. Cox proportional hazards regression models were used to calculate hazard ratios (HRs) of vertebral fractures among patients according to thiazide or loop diuretic use within 2 years following stroke. Sensitivity analyses based on the duration of thiazide or loop diuretic use were further conducted.

**Results:**

There was no significant difference in vertebral fracture risk between thiazide users and non-users (adjusted HR [aHR] = 1.14, 95% confidence interval [CI] = 0.88–1.47, *p* = 0.316). Loop diuretic users had a significantly higher vertebral fracture risk than non-users (aHR = 1.45, 95% CI = 1.06–1.98, *p* = 0.019). However, the sensitivity analysis revealed that short-term thiazide use (exposure duration < 90 days within 2 years after stroke) significantly increased the risk of vertebral fracture versus non-use (aHR = 1.38, 95% CI = 1.02–1.88, *p* = 0.039). Only short-term loop diuretic users had significantly higher risk of vertebral fracture (aHR = 1.56, 95% CI = 1.11–2.20, *p* = 0.011). The other two subgroups with longer exposure duration in analyses for both thiazides and loop diuretics revealed no significant effect.

**Conclusions:**

Short-term thiazide or loop diuretic use was associated with an increased risk of vertebral fracture after stroke. Further prospective clinical trials are required to confirm this finding.

## Background

The most common sites of osteoporotic fracture are the vertebrae [1]. Previous research illustrated that the overall prevalence of vertebral fracture was approximately 13%, including prevalence as high as 20% in people aged ≥70 years [2]. Vertebral fracture is associated with significant disability, morbidity, and mortality [3–5]. Stroke is a major risk factor for osteoporosis and fracture [6]. Previous studies indicated that bone mineral density (BMD) remarkably decreased soon after stroke [7, 8]. Both decline in BMD and increase in the risk of falls elevate the risk of fractures following stroke [9, 10].

Hypertension is a very common comorbidity observed in patients with stroke. Previous studies revealed that 79% of ischemic stroke patients and 85% of hemorrhagic stroke patients have hypertension as a comorbidity [11, 12]. Both thiazides and loop diuretics have been widely used for the treatment of hypertension [13–15]. Thiazides, in addition to reducing blood pressure, can modulate calcium homeostasis, thus effectively preserve BMD [16]. Previous studies revealed that thiazides not only reduce urinary calcium excretion but also directly stimulate osteoblast differentiation and bone mineral formation [17]. However, thiazides can also induce hyponatremia, which was suggested by several studies to be associated with increased fracture risk [18–21]. Loop diuretics were reported to increase urinary calcium excretion [22], and they could theoretically lower BMD, which is a known risk factor for osteoporotic fracture. However, the results concerning the association of loop diuretics and BMD were inconsistent among studies reported [23–25]. In addition, loop diuretics may potentially increase the risk of falls [26].

Several studies evaluated the association of diuretic use and hip fracture risk [27–32]. However, only a few studies have evaluated the vertebral fracture risk [33–37], although vertebral fracture was the most common type of osteoporotic fracture, and it can also cause significant morbidity and mortality, and a considerable socioeconomic burden. Moreover, no current study on this issue is based on post-stroke patients, which is a specific population prone to developing fracture due to declining BMD and an increased risk of falls as mentioned above. The evidence on whether thiazide or loop diuretic use influences the risk of vertebral fracture in post-stroke patients is still insufficient. Therefore, we conducted a population-based propensity score-matched retrospective cohort study to investigate whether thiazide or loop diuretic use affected the risk of vertebral fracture after stroke.

## Methods

### Data sources

The data of this present study were obtained from the Taiwan National Health Insurance Research Database. In 1995, Taiwan began a National Health Insurance (NHI) program, which is a single-payer program administered by the government, to finance health care for all residents in Taiwan. Approximately 99% of the Taiwanese population was covered and 97% of the hospitals and clinics had contracts with the NHI program in Taiwan [38, 39]. We analyzed a representative database of 1000,000 people, called the Longitudinal Heath Insurance Database (LHID), which was randomly sampled from all NHI beneficiaries in the registry of year 2000 in Taiwan by the National Health Research Institute (NHRI) for research purposes. The LHID included comprehensive information on the sampled insured people, such as demographic data, dates of clinical visits or hospitalization, diagnostic codes, expenditure amounts, and details of prescriptions [40]. The NHRI reported no statistically significant difference in age, sex, or medical costs between the sampling cohort in LHID and all NHI beneficiaries in Taiwan [41]. After receiving NHRI approval, we used the LHID to conduct our study. Personal identification information was encrypted before releasing the research database from the NHRI to protect patient privacy and data security. This study was approved by the Institutional Review Board of Tzu Chi Medical Center.

### Study population

All patients diagnosed with new-onset stroke between January 2000 and December 2011 comprised the study population. As our previous study also focused on stroke patients [42], we used the primary discharge diagnosis of stroke (International Classification of Diseases, 9th Revision, Clinical Modification [ICD-9-CM] codes 430–437) to identify these patients. The index date was defined as the date of stroke diagnosis, and index hospitalization was defined as hospitalization for new-onset stroke.

As our previous study evaluated the association between thiazides and hip fracture [43], we retrieved all prescription data for thiazides and loop diuretics within 2 years after the diagnosis of new-onset stroke to evaluate the association between diuretic use and the risk of vertebral fractures. Patients who were prescribed thiazides within 2 years after stroke were defined as the thiazide cohort, those who were prescribed loop diuretics comprised the loop diuretic cohort, and those who had not been prescribed any thiazides or loop diuretics were defined as the comparison cohort. To further evaluate the effects of short- and long-term exposure to diuretics, both the thiazide and loop diuretic cohorts were divided into three subgroups [43]: patients with exposure duration of 1–90, 91–365, and > 365 days within 2 years following stroke.

**Fig. 1:**
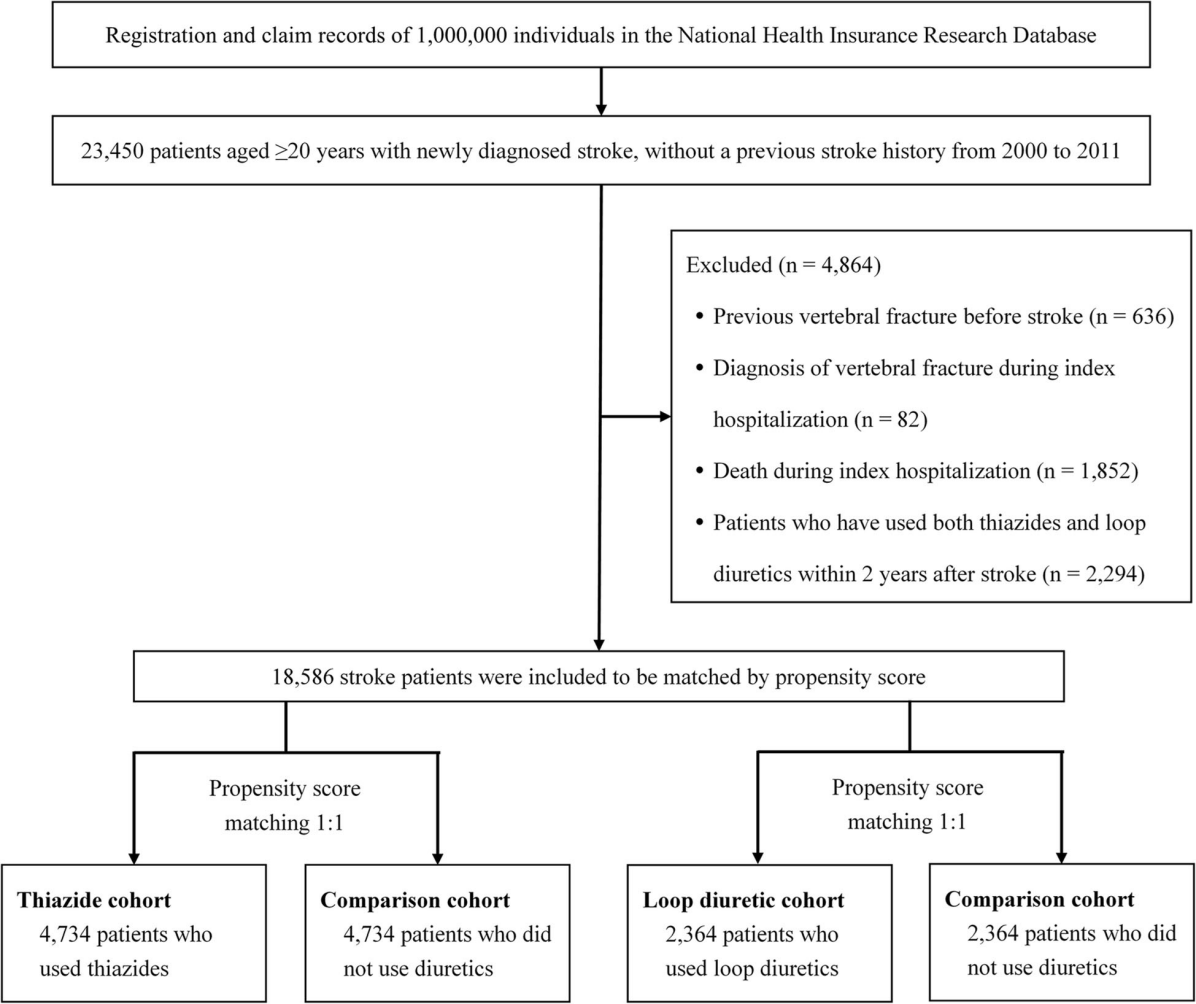
Flow diagram of the enrollment of the study subjects

The exclusion criteria were 1) age < 20 years; 2) history of stroke before the study period of 2000–2011; 3) history of previous vertebral fracture before stroke; 4) concurrent diagnosis of vertebral fracture during index hospitalization; 5) death during index hospitalization, and 6) use of both thiazides and loop diuretics within 2 years after stroke (Fig. 1).

### Primary outcome and covariates

The ICD-9-CM codes 733.13, 805.x, and 806.x were used to identify inpatients and outpatients who developed vertebral fractures within 2 years after stroke. All the study subjects were followed up from index date until a new diagnosis of vertebral fracture, withdrawal from the LHID database, or the end of the 2-year follow-up period after stroke. A previous research validated the ICD-9-CM codes for bone fractures, including vertebral fracture, and confirmed their high accuracy [44].

Baseline co-morbidities were identified according to ICD-9-CM codes, including hypertension, diabetes mellitus, chronic obstructive pulmonary disease, congestive heart failure, chronic liver disease, chronic kidney disease, osteoporosis, malignancy, dementia, depression, epilepsy, and parkinsonism. The overall Charlson comorbidity index was also calculated [45]. The type of Stroke was assigned as per the ICD-9-CM codes for the primary diagnosis during index hospitalization.

The socioeconomic status of the study subjects was approximated using insurance premium and urbanization levels. Insurance premiums, which served as a proxy for income level, were grouped into four categories (≥$40,000, $20,000–39,999, $1–19,999, and fixed amount [New Taiwan dollars]). The incomes of people without salaries such as students, children, elderly, and the unemployed were designated as “fixed amount” (financial dependents), and their insurance premiums were covered by the government or their foster families [46]. Based on information in the NHI research database, subjects were also stratified by residence. Urbanization in Taiwan was categorized into seven levels [47, 48], with a lower level indicating a higher degree of urbanization. Levels 5 through 7 were combined into a single group to simplify the analysis [43].

### Statistical analysis

To minimize selection bias, we performed propensity score matching to balance all baseline characteristics listed in Table 1. To analyze the effect of thiazides, we matched patients who received thiazides (thiazide cohort) to those who did not receive diuretics (comparison cohort) in a 1:1 ratio. To analyze the effect of loop diuretics, we matched patients who received loop diuretics (loop diuretic cohort) to those who did not receive diuretics (comparison cohort) in the same ratio. Propensity score matching was conducted without replacement, and the nearest-neighbor algorithm was applied to construct matched pairs.

**Table 1:** Baseline characteristics of patients with stroke according to thiazide use after propensity score matching

	Thiazide use				*p* value
	Yes (*n* = 4734)		No (*n* = 4734)		
	n	%	n	%	
Age (years)					0.206
< 60	1454	30.7	1411	29.8	
60–79	2601	54.9	2684	56.7	
≥ 80	679	14.3	639	13.5	
Gender					0.661
Male	2776	58.6	2755	58.2	
Female	1958	41.4	1979	41.8	
Income level (NTD)					0.737
Financially dependent	1281	27.1	1312	27.7	
1–19,999	2466	52.1	2464	52.1	
20,000–39,999	646	13.7	641	13.5	
≥ 40,000	341	7.2	317	6.7	
Urbanization level					0.809
1 (Most urbanized)	1138	24.0	1148	24.3	
2	1242	26.2	1271	26.9	
3	860	18.2	852	18.0	
4	848	17.9	855	18.1	
5 (Least urbanized)	646	13.7	608	12.8	
Stroke type					0.918
Ischemic	2861	60.4	2864	60.5	
Hemorrhagic	807	17.1	821	17.3	
TIA	617	13.0	596	12.6	
Others	449	9.5	453	9.6	
Charlson comorbidity index					0.707
1–2	2936	62.0	2957	62.5	
3–4	1338	28.3	1304	27.5	
≥ 5	460	9.7	473	10.0	
Hypertension	4268	90.2	4267	90.1	0.972
Diabetes mellitus	1678	35.5	1663	35.1	0.747
COPD	790	16.7	795	16.8	0.891
Congestive heart failure	290	6.1	298	6.3	0.733
Chronic liver disease	417	8.8	432	9.1	0.590
Chronic kidney disease	293	6.2	294	6.2	0.966
Osteoporosis	206	4.4	215	4.5	0.654
Malignancy	214	4.5	216	4.6	0.921
Dementia	202	4.3	207	4.4	0.800
Depression	194	4.1	191	4.0	0.876
Epilepsy	63	1.3	66	1.4	0.790
Parkinsonism	126	2.7	116	2.5	0.515

A χ2 test was used to compare the differences between the two groups

Abbreviations: *NTD* New Taiwan dollars, *COPD* chronic obstructive pulmonary disease, *TIA* transient ischemic attack

A χ2 analysis was used to analyze categorical variables in this study. The vertebral fracture-free rates were estimated using the Kaplan-Meier method, and the difference between these survival curves was analyzed using the log-rank test. Univariate and multivariate Cox proportional hazards regression models were used to calculate the incidence rate, hazard ratios (HRs), and 95% confidence intervals (CIs) of vertebral fracture risk in association with thiazides or loop diuretics. All baseline characteristics listed in Table 1 were adjusted when performing multivariate Cox proportional hazards regression model. Sensitivity analyses were performed to examine whether different exposure durations for thiazides or loop diuretics affected the outcomes. A probability value of < 0.05 was considered statistically significant. Stata version 13 (Stata Corporation, College Station, Texas, USA) was used to perform all analyses.

### Ethics approval and consent to participate

As the present study used de-identified secondary data, the patients were not directly involved in this study and thus the need for consent was waived. This study was approved by the Tzu-Chi General Hospital Research Ethics Committee (REC No. IRB107–05-C).

## Results

### Patient characteristics

After excluding patients who did not meet the study criteria and performing propensity score matching, 4734 thiazide users were identified as the thiazide cohort, and 4734 patients who did not use diuretics were matched as the comparison cohort to analyze the effects of thiazides. To analyze the effect of loop diuretics, 2364 loop diuretic users were identified as the loop diuretic cohort, and 2364 non-users comprised the comparison cohort (Fig. 1). There were no significant differences between the groups for all baseline characteristics including age, gender, income level, urbanization level, stroke type, Charlson comorbidity index, and comorbidities after propensity score matching (Table 1 for thiazide use and Table 2 for loop diuretic use).

**Table 2:** Baseline characteristics of patients with stroke according to loop diuretic use after propensity score matching

	Loop diuretic use				*p* value
	Yes (*n* = 2364)		No (*n* = 2364)		
	n	%	n	%	
Age (years)					0.645
< 60	398	16.8	375	15.9	
60–79	1306	55.3	1314	55.6	
≥ 80	660	27.9	675	28.5	
Gender					0.792
Male	1344	56.9	1335	56.5	
Female	1020	43.1	1029	43.5	
Income level (NTD)					0.215
Financially dependent	822	34.8	821	34.7	
1–19,999	1283	54.3	1323	56.0	
20,000–39,999	188	7.9	167	7.1	
≥ 40,000	71	3.0	53	2.2	
Urbanization level					0.943
1 (Most urbanized)	542	22.9	541	22.9	
2	604	25.6	612	25.9	
3	403	17.1	402	17.0	
4	469	19.8	449	19.0	
5 (Least urbanized)	346	14.6	360	15.2	
Stroke type					0.524
Ischemic	1431	60.5	1467	62.0	
Hemorrhagic	373	15.8	380	16.1	
TIA	321	13.6	298	12.6	
Others	239	10.1	219	9.3	
Charlson comorbidity index					0.415
1–2	1073	45.4	1110	47.0	
3–4	774	32.7	733	31.0	
≥ 5	517	21.9	521	22.0	
Hypertension	1840	77.8	1854	78.4	0.622
Diabetes mellitus	970	41.0	976	41.3	0.859
COPD	646	27.3	657	27.8	0.720
Congestive heart failure	459	19.4	427	18.1	0.233
Chronic liver disease	299	12.7	288	12.2	0.628
Chronic kidney disease	379	16.0	355	15.0	0.335
Osteoporosis	133	5.6	133	5.6	1.000
Malignancy	190	8.0	170	7.2	0.273
Dementia	169	7.1	154	6.5	0.387
Depression	139	5.9	128	5.4	0.488
Epilepsy	46	1.9	39	1.6	0.444
Parkinsonism	121	5.1	122	5.2	0.947

A χ2 test was used to compare the differences between the two groups

Abbreviations: *NTD* New Taiwan dollars, *COPD* chronic obstructive pulmonary disease, *TIA* transient ischemic attack

### Thiazide use and vertebral fracture risk

**Fig. 2:**
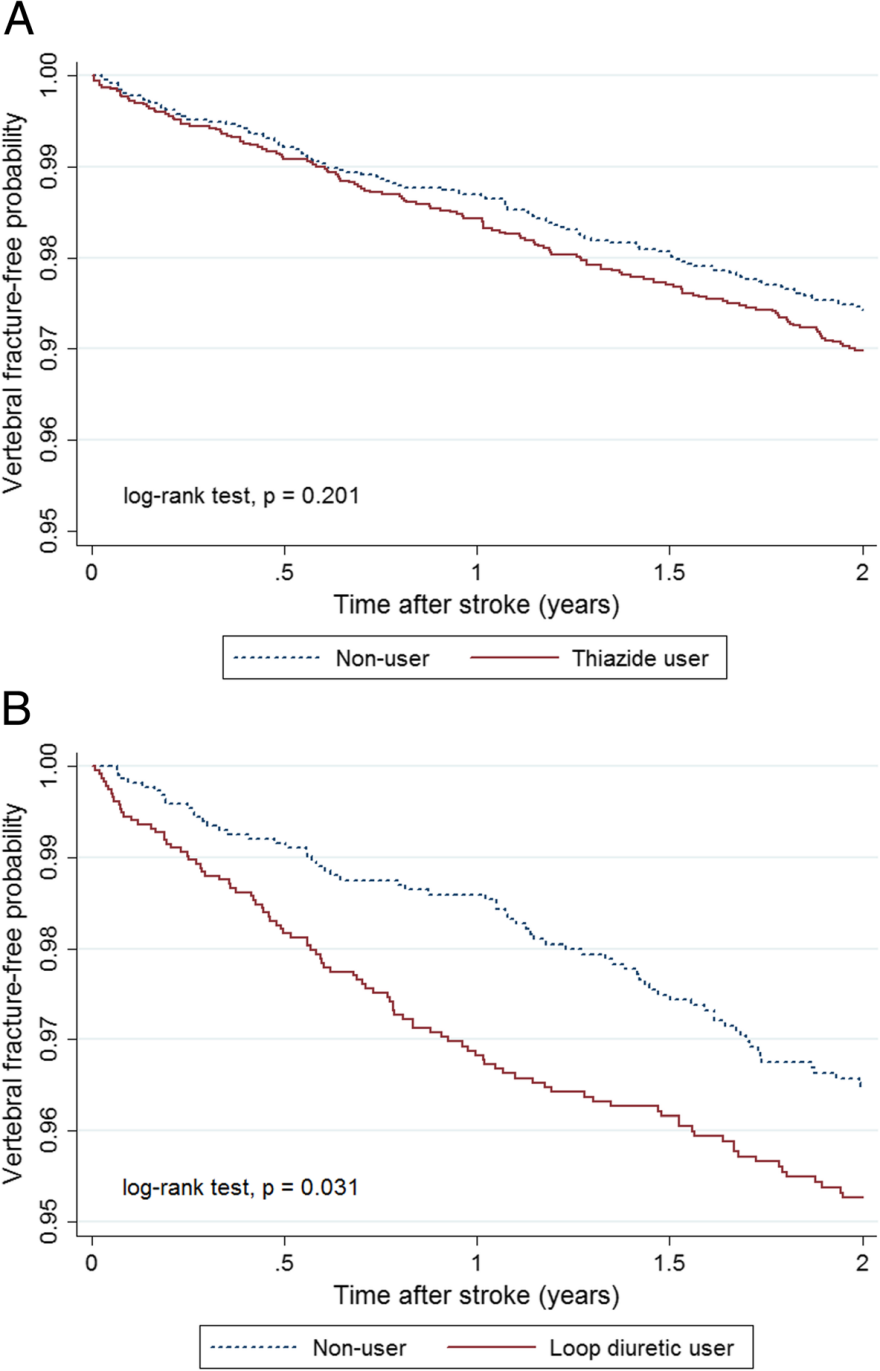
Kaplan–Meier curves showing estimated vertebral fracture-free probability in post-stroke patients according to diuretic use. **a** Thiazide user vs. non-user. **b** Loop diuretic user vs. non-user

Kaplan–Meier survival analysis revealed no significant difference in the cumulative incidence of vertebral fracture between the thiazide and comparison cohorts (15.4 per 1000 person-years vs. 13.1 per 1000 person-years, *p* = 0.201) (Fig. 2a). The HRs of vertebral fracture associated with thiazide use were not significantly different in both univariate (crude HR = 1.18, 95% CI = 0.92–1.52, p = 0.201) and multivariate Cox proportional hazards regression models (adjusted HR = 1.14, 95% CI = 0.88–1.47, *p* = 0.316) (Table 3).

**Table 3:** Risk of vertebral fracture for patients with stroke according to thiazide and loop diuretic use

	Thiazide use		Loop diuretic use	
	Yes (*n* = 4734)	No (*n* = 4734)	Yes (*n* = 2364)	No (*n* = 2364)
Cases of vertebral fracture	138	108	99	67
Person-years	8975.9	8251.3	3928.8	3740.5
Incidence rate^a^	15.4	13.1	25.2	17.9
Univariate model				
crude HR (95% CI)	1.18 (0.92–1.52)	1 (ref.)	1.40 (1.03–1.92)	1 (ref.)
*p* value	0.201		0.032	
Multivariate model^b^				
adjusted HR (95% CI)	1.14 (0.88–1.47)	1 (ref.)	1.45 (1.06–1.98)	1 (ref.)
*p* value	0.316		0.019	

^a^Per 1000 person-years

^b^Multivariate Cox proportional hazard regression model with adjustment for all baseline characteristics shown in Table 1

Abbreviations: *HR* hazard ratio, *CI* confidence interval

Sensitivity analyses to evaluate the effect of exposure duration within 2 years after stroke revealed that only 1–90 days of thiazide use significantly increased the risk of vertebral fracture compared with non-use (adjusted HR = 1.38, 95% CI = 1.02–1.88, *p* = 0.039), whereas longer exposure durations had no significant effect. In addition, we observed a trend that estimates of HRs for vertebral fracture tended to be lower as the exposure duration increased (Table 4).

**Table 4:** Adjusted HRs of vertebral fracture according to the duration of use of thiazides and loop diuretics

Duration of use	Thiazides			Loop diuretics		
	adjusted HR^a^	95% CI	*p* value	adjusted HR^a^	95% CI	*p* value
None	1 (ref.)			1 (ref.)		
1–90 days	1.38	1.02–1.88	0.039	1.56	1.11–2.20	0.011
91–365 days	1.14	0.80–1.62	0.470	1.32	0.81–2.14	0.263
> 365 days	0.80	0.53–1.22	0.298	1.23	0.66–2.29	0.521

^a^Calculated using a multivariate Cox proportional hazard regression model with adjustment for all baseline characteristics shown in Table 1

Abbreviations: *HR* hazard ratio, *CI* confidence interval

### Loop diuretic use and vertebral fracture risk

The cumulative incidence of vertebral fracture in the loop diuretic cohort was significantly higher than that in the comparison cohort (25.2 per 1000 person-years vs. 17.9 per 1000 person-years, *p* = 0.031) (Fig. 2b). The HRs also revealed that the loop diuretic cohort had a significantly higher risk of vertebral fracture than the comparison cohort in both univariate (crude HR = 1.40, 95% CI = 1.03–1.92, *p* = 0.032) and multivariate Cox proportional hazards regression models (adjusted HR = 1.45, 95% CI = 1.06–1.98, *p* = 0.019) (Table 3).

Sensitivity analyses for the exposure duration revealed that only 1–90 days of loop diuretic use significantly increased the risk of vertebral fracture compared with non-use (adjusted HR = 1.56, 95% CI = 1.11–2.20, *p* = 0.011). The estimates of HRs for vertebral fracture appeared to decrease as the exposure duration increased, and no significant effect was observed in the two subgroups with longer exposure durations (Table 4).

## Discussion

In this population-based propensity score-matched retrospective cohort study, we found that short-term use of thiazides or loop diuretics after stroke was associated with an increased risk of vertebral fracture. To the best of our knowledge, this is the first study to investigate the association between diuretic use and the risk of vertebral fracture after stroke. Vertebral fracture risk is an important issue to be addressed among patients who use diuretics after stroke because many of these patients receive concurrent hypertension treatment, and stroke is a major risk factor for osteoporosis and fracture [6]. However, the relevant literature was sparse.

The higher vertebral fracture risk associated with thiazide use after stroke was unexpected, although the effect was only linked to short-term use. Previous studies have suggested that thiazide diuretics decrease hip fracture risk [27, 28]. Few studies have evaluated vertebral fractures, and the results were inconsistent [33, 34, 37]. One previous study, which only included women ≥65 years of age, demonstrated that thiazide use was not identified as a risk or protective factor of vertebral fracture [33]. Another large-scale case-control study in Denmark, which focused on the general population, found that use of thiazides is associated with significantly reduced risks of all fractures, but non-significant results were found for vertebral fractures [34]. However, on sub-analyses after categorizing patients according to cumulative doses, the aforementioned study found a significant higher risk of vertebral fracture in patients who received low cumulative doses of thiazides. The other two higher-dose categories showed no significant association between thiazide use and vertebral fracture risks [34]. The results seem more compatible with our study results, which revealed that only short-term thiazide use was associated with increased vertebral fracture risks, but such association was not found in those with longer-duration thiazide use. In addition, the other recent large-scale cohort study, which only included female patients, revealed that thiazides and loop diuretics are independently associated with an increased risk of vertebral fracture [37], but this study only categorized subjects into binary categories of diuretic use through questionnaires, without examining the effect of the exposure duration or dose.

Among stroke patients, diuretics were commonly prescribed owing to the high prevalence of hypertension. Stroke patients are at greater risks of developing fracture, and fractures after stroke contribute to further decreased recovery of independent mobility and might cause disability, morbidity, and mortality. Our study, which focused on stroke patients, included homogeneous study subjects and more precise evidence for such a unique and important population. Moreover, our study results clearly indicated only short-term, not long-term, use of thiazides and loop diuretics, which is associated with higher risks of vertebral fractures. This information may cause clinicians and patients to consider and weigh the possible benefits and harmful effects before prescribing diuretics in stroke survivors.

A possible explanation for the increased vertebral fracture risk among thiazide users could be hyponatremia [37], which reportedly develops in approximately 30% of thiazide users [49]. Thiazide-induced hyponatremia occurred rapidly, appearing within a mean of 19 days after starting treatment [50]. Hyponatremia, even if only mild, might increase the risk of falls and consequent fractures due to gait and attention impairments [51, 52]. In addition, studies revealed that hyponatremia can have direct effects on bone via the activation of osteoclasts [53], which may cause microdamage of bone, resulting in decreased bone quality [54], and a further increased risk of osteoporosis and fracture of the spine because it is particularly susceptible to microdamage relative to other fracture sites [18, 37, 55, 56]. However, the sensitivity analyses in this present study revealed no such effect of increased fracture risk in the two subgroups with longer thiazide exposure durations. We should not ignore that thiazides can also modulate calcium homeostasis, which is effective in preserving BMD [16]. However, the effects on BMD take a long time to appear. One study conducted by Bolland et al. evaluated the association of thiazide use and BMD in postmenopausal women, and the study indicated that the protective effect of thiazides on BMD appeared 6–12 months after treatment initiation [57]. In addition, another study conducted by Kruse et al. indicated that thiazide use was found to increase fracture risk during weeks 1–42 of treatment and then gradually decrease the risk starting from week 43, and this study concluded that a long duration of continuous thiazide exposure is important for obtaining a protective effect on fracture risk [58]. These findings could possibly explain our observation that only short-term thiazide users had higher vertebral fracture risk. When the exposure duration increased, the estimated HRs of vertebral fracture decreased, as shown in Table 4.

The findings of associations between loop diuretic use and increased vertebral fracture risk were expected. However, this is the first study to evaluate the effect in patients who experienced a stroke. The use of loop diuretics was found to increase fracture risk due to several reasons, as mentioned in previous studies. Loop diuretics increased urinary calcium excretion, and they might have promoted higher rates of bone loss and bone porosity [59, 60]. The drugs might have also potentially caused orthostatic hypotension and fall [26, 61], subsequently increasing the risk of fractures [60].

However, the sensitivity analyses revealed that only short-term loop diuretic users had a significantly higher vertebral fracture risk. No such significant effect was found in the two subgroups with longer exposure durations. A previous study focusing on long-term loop diuretic users indicated that increased renal calcium loss after using loop diuretics is compensated by a parathyroid hormone-dependent increase in 1,25-dihydroxyvitamin D levels. Surprisingly, the exposure duration of loop diuretics was positively associated with spinal BMD, which increased by 0.3% per year of loop diuretic treatment [23]. In addition, Berry et al. indicated that an increased risk of fracture was observed only during the initiation period of diuretics, including both thiazides and loop diuretics, although the study outcome was based on a case of hip fracture. The authors concluded that the hip fracture risk was only transiently elevated after starting the use of loop or thiazide diuretics [30]. Therefore, in addition to the aforementioned reasons, these findings can also explain why only patients with short-term diuretic exposure within 2 years after stroke had a higher fracture risk in analyses of both thiazides and loop diuretics in this present study.

The present study was based on a nationwide population-based design, as all results originated from a representative sample of 1 million subjects randomly selected from the NHI research database in Taiwan. The database provided a sufficient sample size to evaluate the effect of diuretics on vertebral fracture risk, specifically in post-stroke patients. Nevertheless, our study has several limitations that should be mentioned. First, we could not retrieve some clinical data, including patient lifestyles and physical, psychiatric, or laboratory examination data (e.g., BMD, body mass index, smoking status, and alcohol use) from the NHI research database [43], and they might represent confounding factors of fracture risk. Even if we had an adequate study design, including propensity score matching to balance baseline differences between groups and further use of a multivariate Cox proportional hazards regression model to eliminate residual confounding effects, bias related to unknown or unmeasured confounders might still exist. Second, because of using the de-identified secondary data, we could not directly approach the patients to monitor the exact intake of diuretics or other medications. We also could not identify the exact mechanism of how the vertebral fracture occurred to distinguish whether the fracture was caused by osteoporosis or a trauma event. Third, because of the patient anonymity policy in the NHI research database, we could not confirm the accuracy of the patients’ diagnoses by contacting the patients directly [43]. We also could not obtain the detailed image reports in the claims-based dataset; thus, we could not determine what types of image and how such image examination confirmed the vertebral fracture. However, the subjects who developed stroke were identified only when they were hospitalized with a primary diagnosis of stroke. In addition, the ICD-9-CM codes for the diagnosis of vertebral fracture were validated in a previous study, and they displayed high accuracy in both inpatients and outpatient service [44]. Moreover, hospitals or physicians in Taiwan would be heavily fined for incorrect diagnoses or coding [62]. Thus, the validity of the diagnoses of stroke and vertebral fracture should be considered acceptable.

## Conclusions

In summary, this population-based propensity score-matched retrospective cohort study indicated that short-term thiazide or loop diuretic treatment was associated with an increased risk of vertebral fracture in post-stroke patients. Patients and clinicians should keep this association in mind and consider and weigh the possible benefits and harm of diuretics before prescribing diuretics in stroke survivors. Further prospective clinical trials are required to confirm this finding.

## Abbreviations

BMD: Bone mineral density
CI: Confidence interval
COPD: Chronic obstructive pulmonary disease
HR: Hazard ratio
ICD-9-CM: International Classification of Diseases, 9th Revision, Clinical Modification
LHID: Longitudinal Heath Insurance Database
NHI: National Health Insurance
NHRI: National Health Research Institute
TIA: Transient ischemic attack
